# Aetiology of lateral progression of arthritis following Oxford medial unicompartmental knee replacement: a case–control study

**DOI:** 10.1007/s12306-015-0394-8

**Published:** 2016-01-25

**Authors:** H. Pandit, B. Spiegelberg, A. Clavé, C. McGrath, A. D. Liddle, D. W. Murray

**Affiliations:** 1 The Nuffield Department of Orthopaedics, Rheumatology and Musculoskeletal Sciences (NDORMS), University of Oxford, Headington, Oxford, OX3 7LD UK; 2 The Nuffield Orthopaedic Centre (NOC), Oxford University Hospital NHS Trust, University of Oxford, Headington, Oxford, OX3 7LD UK

**Keywords:** Unicompartmental knee replacement, Lateral osteoarthritis, BMI, Component position

## Abstract

**Purpose:**

The aim of this case–control study is to assess for predictive factors that may determine development of lateral compartment progression after Oxford medial unicompartmental knee replacement.

**Methods:**

Twenty-eight patients who were revised as a result of lateral osteoarthritis progression were matched to 52 alive and unrevised patients. Body mass index, intra-operative findings, postoperative leg alignment, meniscal bearing size and histological findings have been analysed. Radiological analysis was carried out on the immediate postoperative radiographs by two blinded observers to assess the severity of arthritis in the lateral compartment. The measurements of the components positions were converted into binary figures as to whether they were inside or outside the recommended limits for analysis. Conditional logistic regression was used to identify important predictors of progression, taking into account the case–control grouping.

**Results:**

The results shows that the condition of the lateral compartment is a significant predictor for developing subsequent lateral compartment arthrosis (OR 2.627, *p* = 0.019). The study showed no relationship between progression of arthritis and component position (OR [0.5–1.18], *p* [0.21–1]). Nor have it demonstrated that BMI (OR 1.06, *p* = 0.61), postoperative leg alignment (OR 1.26, *p* = 0.636), meniscal bearing size (1.32, *p* = 0.307) or presence of chondrocalcinosis (OR 0.35, *p* = 0.36) have any association with lateral osteoarthritis progression.

**Conclusions:**

This study showed the importance of excluding radiographic evidence of lateral compartment osteoarthritis on the preoperative radiograph prior to medial unicompartmental knee replacement. We have not been able to show any relationship between progression of arthritis and component position.

*Level of proof* Case–control study, level III.

## Introduction

The Oxford^®^ unicompartmental knee replacement (Biomet, Bridgend, UK) is indicated for use in patients with symptomatic end-stage medial compartment osteoarthritis. It incorporates a fully mobile meniscal bearing that minimises linear wear [1] and has long-term data that demonstrate good results both in terms of functional outcome and survivorship [2–4].

In the latest National Joint Registry (NJR), lateral progression of arthritis is the third most common reason for further surgical intervention following a unicompartmental knee replacement [4]. Progressive osteoarthritis was the cause of failure in 25–34 % of cases in an early series [5] often within the first 5 years. Although these rates have fallen significantly with improvements in design, surgical technique and patient selection, in more recent cohort studies lateral progression of osteoarthritis is still a common cause for revision ranging from 0.9 to 7 % [2, 6–8].

It has been suggested that arthritis progression is the result of errors of implantation, particularly over-correction of the varus deformity associated with anteromedial arthritis causing lateral compartment overload. However, there is little difference in the comparison rates of failure from lateral progression in series from high-volume centres, series from less experienced surgeons and NJR data [4]. This suggests that the aetiology for arthritis progression may be more complex than simply being the direct result of technical errors.

The aim of this study is to assess for predictive factors that may determine development of lateral compartment progression.

## Patients and methods

Between January 1998 and November 2011, 2333 knees (1899 patients) underwent an Oxford unicompartmental knee replacement for anteromedial osteoarthritis. The details of their surgery were recorded prospectively and stored in an arthroplasty database according to ethical directives. From this database, 28 consecutive patients who were revised as a direct result of progression of arthritis into the lateral compartment were selected as the case group. Two cases with revision due to lateral progression were excluded; one had previously undergone anterior cruciate ligament (ACL) reconstruction and in the other case the original postoperative radiographs were missing. Following identification of these cases, all remaining alive and unrevised patients within the same database were screened for inclusion within the control group. An optimal matching algorithm was used to identify the closest matches on the basis of age, gender, body mass index (BMI) and time since surgery. If more than two potential matches were identified, two patients were picked at random from the pool of potential matches. All of these patients had a well functioning Oxford medial UKR with no evidence of arthritis progression. Each control patient remained alive with their index implant in situ for at least the same amount of time as the case patient before their revision operation. A total of 78 patients were selected, 38 females and 40 males.

All patients underwent a standardised phase 3 OUKR procedure in the same centre. The patients were selected for surgery based on the principles given by Goodfellow et al. [9]. There should be full thickness cartilage loss affecting both tibia and femur on the medial side with preservation of full thickness cartilage in the lateral compartment, and this was assessed on varus/valgus stress radiographs. The medial collateral ligament (MCL) should be functionally normal, as demonstrated by a correctable intra-articular varus deformity in 20° of flexion, best shown on valgus stress view. The ACL should be functionally intact. The lateral compartment was checked intra-operatively to confirm the medial OUKR indication. The presence of a chondral ulcer on the medial side of the lateral femoral condyle can be ignored [10]. Presence of arthritic changes within the patellofemoral joint provided there is not severe lateral patella facet OA with bone loss and subluxation can also be ignored [11, 12]. A Patient’s age, weight, level of activity and presence of chondrocalcinosis is not considered to be a contraindication [13].

Patient-specific data were collected including height and weight from which a BMI was calculated. Leg alignment was measured using a longarm goniometer, and all relevant surgical findings (as ACL deficiency or alteration in the lateral compartment) were assessed intra-operatively and carefully collected on a standardised operative form.

### Radiological assessment

Standardised weight-bearing postoperative radiographs were taken using a digital radiology system. The anteroposterior (AP) radiographs were aligned with the tibial tray, and the lateral radiographs were aligned such that the femoral condyles were superimposed. The immediate postoperative radiographs were reviewed by two observers (BGIS, AL) to assess the severity of arthritis in the retained lateral compartment on AP view using the Kellgren and Lawrence classification [14]; (0 = normal, 1 = possible osteophyte, no joint space narrowing [JSN], 2 = definite osteophyte, possible JSN, 3 = multiple osteophytes, definite JSN, 4 = large osteophytes, marked JSN, severe sclerosis). The observers were blinded as to which group the patient was in and the grade was determined by consensus, as described in the original paper [14]. Each radiograph was assessed twice by the same observer using the method described by Weale et al. [8].

**Fig. 1:**
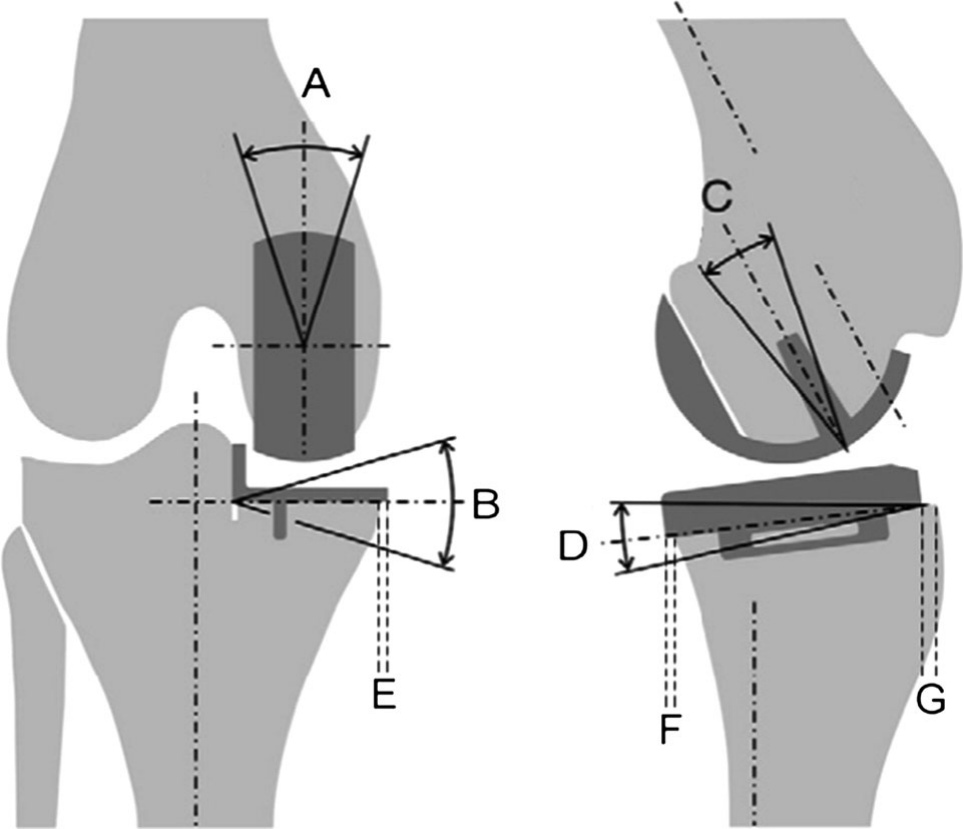
Measurements obtained from radiological analysis. *A* Femoral component valgus/varus. *B* Tibial component valgus/varus. *C* Femoral component flexion/extension. *D* Tibial component tilt. *E* Tibial component medial overhang. *F* Tibial component anterior overhang. *G* Tibial component posterior overhang

Bespoke image analysis software (MATLAB, The MathWorks Inc. Natick, MA, USA) was used to assess component position on the postoperative radiographs. From the anteroposterior radiograph, coronal malalignment of the implants could be measured and also the extent of medial overhang of the tibial plate. From the lateral view, femoral flexion, tibial tilt and anteroposterior tibial overhang were measured (Fig. 1). These measurements were taken twice by two different observers who were both blinded as to whether the patients were case or control. The measurements were converted into binary figures as to whether they were inside or outside the recommended limits as described by Goodfellow et al. [15].

### Statistical analysis

The categorical values are given in percentage, and the quantitative values are given in mean and standard deviation (SD). Conditional logistic regression was used to identify important predictors of progression, taking into account the case–control grouping. Univariable and multivariable analyses were performed. The radiographic measurements all have the potential to deviate from normal in one of two directions (e.g., valgus and varus), and as a result, the effect of each of these measurements on probability of progression are nonlinear. These predictors were therefore categorised into measures inside and outside the acceptable limits for implant position. In this way, the data are analysed as dichotomous variables. The inter- and intra-observer errors for implant alignment were assessed using an intraclass correlation coefficient. All statistical analyses were performed using Stata IC v.12.1 (Stata Corp., College Station, TX, USA).

## Results

Twenty-six patients made up the case group and 52 the control group. The case group comprised 12 females and 14 males; the mean age at time of surgery was 68.8 (48–81). In the control group, there were 26 females and 26 males; the mean age at time of surgery was 69.3 (49–82). The mean time to revision was 5.1 years (1–10). Full baseline demographics are given in Table 1.

**Table 1:** General demographic features of both case and control groups

	Controls (52)	Cases (26)
Age years (mean and SD)	69.3 ± 8.2	68.8 ± 8.3
Gender (male and %)	27/52 (51.9 %)	14/26 (53.9 %)
BMI kg (SD)	28.8 ± 5.1	28.7 ± 3.8
Time since surgery/years (SD)	10.7 ± 3.0	10.7 ± 2.6
Leg alignment		
Varus	43 (82.7 %)	15 (57.7 %)
Valgus	6 (11.5 %)	0
Neutral	3 (5.8 %)	11 (42.3 %)
Mean° leg alignment (SD)	3.7 ± 3.0 (varus)	3.2 ± 3.0 (varus)
Mean valgus	3.2 ± 1.8	None
Mean varus	4.9 ± 1.1	5.5 ± 1.5
Bearing size		
3	9 (17.3 %)	5 (19.2 %)
4	28 (53.8 %)	11 (42.4 %)
5	13 (25 %)	6 (23.1 %)
6	0	3 (11.5 %)
7 and +	2 (3.9 %)	1 (3.8 %)

During the OUKR procedure, the ACL was reported to be functionally intact for all the patients, both in case and control groups. No critical alterations or anomalies of the lateral compartment were reported.

KL grades for case and control groups are given in Table 2. All five of the patients with the worst scores on immediate postoperative radiographs went on to require revision for arthritis progression.

**Table 2:** Distribution of KL grades between case and control groups

KL grade	Controls (52)	Cases (26)	Total
0	30 (57.7 %)	11 (42.3 %)	41
1	22 (42.3 %)	10 (38.5)	32
2	0	2 (7.7 %)	2
3	0	3 (11.5 %)	3

The inter- and intra-observer measure errors for implant alignment were, respectively, of 0.972 and of 0.96. A result greater than 0.8 is deemed to be good [16].

In regards to implant positioning, the Table 3 shows both groups cases for which the measures were outside the acceptable limits.

**Table 3:** Implants position outside the acceptable limits (*OH* overhang)

	Controls (52)	Cases (26)
Femoral valgus/varus	4	3
Varus >10°	4	2
Valgus >10°	0	1
Femoral flexion/extension	13	14
Flexion >5°	10	9
Extension >5°	3	5
Tibial valgus/varus	1	2
Varus >10°	1	1
Valgus >5°	0	1
Tibial tilt	11	9
Tilt >7°	11	9
Tilt <−5°	0	0
Medial OH >2 mm	6	5
Anterior OH >3 mm	10	6
Posterior OH >2 mm	8	4

Histological analyses were done for 64 patients, and cases group accounts for 24 of them.

Results are shown in Table 4.

**Table 4:** Histological results (*OA* osteoarthritis)

	Controls (40)	Cases (24)
OA	39	20
Mixed	0	2
Chondrocalcinosis	0	1
Inflammatory	0	1
Reactive	1	0

Leg alignment, BMI, bearing size, or any clinical data relating to patient factors did not show significant relationship with lateral compartment progression. Preoperative Kellgren and Lawrence (KL) grade, however, was a significant factor in the development of lateral compartment progression, but the presence of mild radiological lateral compartment osteoarthritis (up to KL 1 or 2) was not associated with an increased probability of revision for progression of disease (Table 5).

**Table 5:** Results of regression analysis excluding alignment positions

Predictor	Odds ratio	95 % CI	*P* value
KL grade	2.627	1.17–5.88	**0.019**
Side	1.25	0.496–3.15	0.636
Bearing size	1.32	0.77–2.28	0.307
BMI	1.06	0.89–1.26	0.61
Chondrocalcinosis	0.35	0.36–3.36	0.36

Significant results are in bold

There was no statistical correlation between component alignment and progression of arthritis (Table 6).

**Table 6:** Results of regression analysis for implant alignment (*OH* overhang)

Predictor	Odds ratio	95 % CI	*P* value
Femoral varus/valgus	0.62	0.12–3.21	0.57
Femoral Flexion	1.24	0.4–3.81	0.71
Tibial varus/valgus	1.56	0.49–4.93	0.45
Tibial slope	0.5	0.17–1.47	0.21
Medial OH	1.18	0.44–3.17	0.74
Anterior OH	1.12	0.35–3.57	0.85
Posterior OH	1	0.33–3.04	1.0

## Discussion

This study shows that the condition of the lateral compartment on the immediate postoperative radiograph is a significant predictor for developing subsequent lateral compartment arthrosis. No other variable was found to have causality.

It is not surprising that cases with evidence of arthritis in the lateral compartment at the time of implantation go on to develop lateral compartment progression. This highlights the importance of a careful clinical and radiographic assessment of the patient prior to surgery. However, despite five patients with the worst KL scores in the lateral compartment subsequently developing arthrosis and needing revision, there are still 21 other cases who developed lateral compartment arthritis despite having normal X-rays. This implies that radiographic findings alone are not the only cause and that lateral compartment progression is multifactorial, and therefore, we have not been able to determine any other associations from the data we have collected.

There was no significant difference between the two groups regarding physiological variables (leg alignment, BMI, chondrocalcinosis status and size of meniscal bearing). It has been suggested that lateral compartment progression may be due to overloading of the lateral compartment due to overcorrection of the varus deformity [17]. For this to happen, a mismatch in the balancing of the knee is required with insertion of an overly large meniscal bearing and impairment of the medial collateral ligament, as an intact MCL would restrain the knee from significant valgus. Our results have not shown any link with either meniscal bearing size or leg alignment. This lack of significance may be attributed to the fact that no knee in the study was considered to be in excess valgus (>10°) as has been previously investigated by Gulati et al. [18].

The BMI in both groups was similarly matched, and it was not found to be associated with a risk of lateral compartment disease progression. There were 20 patients (26 %) who were obese (BMI > 30), 6 of whom were in the case group. It has previously been shown that increasing BMI does not confer an increased risk of failure [19] and this study confirms that.

A deficient ACL is an absolute contraindication for unicompartmental knee replacement [9], and the intact ACL results in the more localised anteromedial osteoarthritis within the medial compartment. The per-operative assessment of ACL’s status at the index operation did not show any difference between both groups.

The presence of chondrocalcinosis within the joint has been thought to indicate an inflammatory process and thereby have an effect on progression of arthritis. This has not been shown to be the case and confirms previous published literature [20]. Histology taken from the medial compartment at the index operation was included for analysis; however, it was not possible to perform regression analysis as the vast majority of the histology specimens were of osteoarthritis.

There were no significant relationships in any of the parameters assessed concerning component positioning in the case or control groups. This suggests that positioning of the Oxford medial unicompartmental knee replacement within the knee has no effect on progression of arthritis within the retained compartment. There is a greater tolerance of component positioning in unicompartmental knees than total knees and more so in the Oxford medial unicompartmental knee as the articulating surface is a fully congruent sphere therefore avoiding non-uniform loading. A recent case series [21] has identified that positioning the femoral component too laterally may result in valgus subsidence of the tibial tray. This is a new phenomenon associated with the cementless OUKR design that was not used in this study.

The limitations to this study are that due to the small sample size, the validity is affected. This is unavoidable as over the timeframe of analysis, there were only 28 implants that had failed as a result of lateral compartment progression; this could be addressed by opening up the study to other centres, but this would reduce the reliability of the results. With the small numbers present, the study is underpowered and a type II error therefore cannot be excluded. The procedures were all carried out in the same centre but are not a single surgeon series. Two surgeons performed 76 % of the control group procedures; however, the same two surgeons only accounted for 52 % of the case group’s procedures, and this may have introduced selection bias into the study but due to small case numbers has been deemed acceptable.

The radiographs used for analysis were of differing levels of quality. True lateral views with the femoral condyles directly superimposed and true anteroposterior views with the tibial component parallel to the X-ray beam were attempted in all cases but inevitably perfection was not achieved in all cases. Therefore, there is some error relating to the radiographic measurements; however, the intra- and inter-observership of the radiographic analysis is good.

This study shows that it is important to exclude radiographic evidence of lateral compartment osteoarthritis on the preoperative radiograph prior to medial unicompartmental knee replacement. We have not been able to show any relationship between progression of arthritis and component position. Nor have we demonstrated that BMI, postoperative leg alignment, meniscal bearing size or presence of chondrocalcinosis have any association with lateral compartment arthritis progression.
